# From redundant association rules to product networks: A scalable confidence-improvement pruning and Maximum Spanning Tree approach

**DOI:** 10.1371/journal.pone.0356990

**Published:** 2026-08-26

**Authors:** Vladimir Đurišić, Saša Vujošević, Ljiljana Kašćelan, Sunčica Vuković

**Affiliations:** Faculty of Economics, University of Montenegro, Podgorica, Montenegro; Shanghai Jiao Tong University, CHINA

## Abstract

Association rule mining is a powerful tool for market basket analysis, yet it frequently produces an overwhelming number of redundant and overlapping rules that hinder practical interpretation. This study presents a scalable hybrid framework that integrates efficient rule reduction with network-based structural analysis to transform large rule sets into concise, actionable product networks. The proposed approach proceeds in two stages. First, association rules are generated using the Apriori algorithm and redundant rules are substantially pruned using a confidence-improvement criterion. The procedure groups rules by their consequent and compares nested antecedent sets, which provides a scalable approximation of structural redundancy detection. Second, the reduced rule set is projected into a weighted product graph using the composite score Lift × Confidence, from which a Maximum Spanning Tree (MaxST) is extracted to identify the highest-scoring non-redundant associations among product categories. The methodology was applied to a large real-world retail dataset from Montenegro comprising approximately 2.64 million basket transactions and 14.9 million product records, aggregated into 46 product groups. The framework achieved a significant reduction in rule volume (e.g., from 403,817–137,186 rules at maxlen = 6) while preserving structurally important relationships. In the baseline maxlen = 3 configuration, the resulting MaxST identified Delicatessen as the dominant hub and uncovered coherent purchasing chains linking delicatessen, beauty and personal care, fresh meat, and healthy produce categories. Results demonstrate that the hybrid method helps bridge the gap between exhaustive rule mining and interpretable network insights, providing retailers with empirically grounded indications for cross-category promotions, bundle design, and shelf-layout testing. The approach combines computational efficiency with managerial relevance, making it suitable for large-scale retail analytics applications.

## 1. Introduction

Association rule mining is one of the most widely used techniques in data-driven retail analytics for uncovering non-obvious dependencies among products and revealing underlying customer purchasing behaviors within large transactional datasets. A key application of this approach is Market Basket Analysis (MBA), which derives actionable insights to support marketing, sales, inventory management, and customer relationship strategies [1,2]. By identifying products that are frequently purchased together, retailers can implement effective cross-selling and up-selling strategies, develop personalized recommendations, optimize store layouts and digital catalogs, improve demand forecasting, and reduce supply chain risks [3,4].

Despite its potential, association rule mining, particularly the Apriori algorithm, frequently generates an excessively large number of rules, many of which are overlapping or redundant, especially in large retail datasets [5]. This redundancy significantly complicates interpretation and diminishes the practical utility of the results for managerial decision-making [6,7]. The core analytical challenge thus shifts from discovering associations to effectively filtering and structuring them so that only the most meaningful, non-redundant patterns are retained.

Previous studies have tackled rule redundancy through simple metric-based filtering or more sophisticated structural methods. Jin et al. [5] introduced a directed hypergraph representation of association rules together with a redundancy-removal procedure based on subordinate and repetitive-path detection. Although theoretically sound, their approach was tested exclusively on small UCI datasets that yielded only 15–18 rules, raising serious questions about its scalability and practical feasibility in real-world retail settings involving millions of transactions.

In parallel, Valle et al. [8] proposed moving beyond exhaustive rule enumeration toward a network-based perspective on product relationships. They modeled products as nodes connected by purchase correlations, transformed these into distance metrics, and extracted minimum spanning trees to identify complements, substitutes, category clusters, and cross-category bridges. However, their method did not perform prior redundancy reduction and relied on correlation-derived distances rather than association-rule strength measures such as lift.

The present study bridges these two complementary research streams by developing a hybrid and scalable methodology. The proposed framework proceeds in two main stages. First, association rules are generated using the Apriori algorithm and redundant rules are substantially reduced through a scalable confidence-improvement pruning procedure based on grouping rules with the same consequent and comparing nested antecedent sets. This procedure enables efficient pruning even in very large transactional datasets without the computational burden of a full directed hypergraph implementation. Second, the reduced rule set is projected into a weighted undirected product graph, with edge weights defined by the composite score Lift × Confidence. A Maximum Spanning Tree (MaxST) is subsequently extracted to retain the highest-scoring non-redundant associations, thereby complementing correlation-based network approaches with an association-rule-based network representation.

This paper makes three principal contributions to MBA:

1. It proposes a scalable confidence-improvement pruning procedure for reducing redundant association rules in large retail datasets. The procedure compares rules with the same consequent and removes expanded antecedents that do not provide a sufficient increase in confidence, thereby improving interpretability while avoiding the computational cost of full structural redundancy analysis.
2. It introduces a novel Lift × Confidence-weighted MaxST that extracts the highest-scoring product associations using association rule-based scoring rather than correlation-based distances.
3. It provides a large-scale empirical validation of the hybrid framework on a real-world retail dataset from Montenegro, consisting of approximately 2.64 million basket transactions and 14.9 million product records aggregated into 46 product groups. This application demonstrates the computational feasibility and interpretability of the proposed approach at a scale rarely addressed in the existing literature.

The remainder of the paper is structured as follows. Section 2 reviews related work on association rule mining, hypergraph-based redundancy reduction, and network-based approaches using spanning trees, highlighting the limitations of prior methods. Section 3 presents the proposed methodology in detail. Section 4 describes the experimental setup, including data preparation and parameter settings. Results are presented in Section 5 and discussed in Section 6. Section 7 addresses the limitations of the study and outlines directions for future research. Section 8 concludes the paper.

## 2. Related work

Market basket analysis is most commonly implemented through association rule mining, with the Apriori algorithm representing one of the foundational methods in this domain [9]. As an unsupervised learning technique, Apriori identifies frequent itemsets in transactional data and derives association rules that describe co-occurrence relationships between products. The strength and relevance of these rules are typically evaluated using support, confidence, and lift, which quantify frequency, conditional probability, and statistical dependence, respectively [10].

Due to its interpretability and direct applicability, association rule mining has been widely adopted in retail analytics. Prior studies demonstrate its effectiveness in supporting recommendation systems, cross-selling strategies, store layout optimization, and category management [11,12]. Segment-specific approaches have further shown that leveraging cross-category dependencies can significantly improve targeted marketing and customer-centric category management [13]. These insights enable retailers to design more effective promotions, optimize inventory and demand forecasting, and enhance overall customer experience.

Despite these advantages, a major limitation of association rule mining emerges in large-scale retail environments. When applied to high-dimensional datasets, algorithms such as Apriori or FP-Growth generate a combinatorial explosion of rules, particularly when support thresholds are lowered to capture less frequent but potentially valuable patterns [6,14]. As a result, the generated rule sets are often characterized by a high degree of redundancy, where many rules represent slight variations of the same underlying relationship. This significantly reduces interpretability and limits the practical usability of the results in decision-making contexts [7,15].

To address this issue, prior research has proposed various rule reduction and pruning techniques. Traditional approaches rely on threshold-based filtering using support, confidence, or lift, aiming to retain only the most significant rules while reducing computational complexity [6]. More advanced methods introduce additional interestingness measures or exploit structural relationships between rules to eliminate redundancy [16,17]. However, these approaches often treat rules as independent entities and do not fully capture the structural dependencies among products.

In response to these limitations, hypergraph-based representations have been proposed as a more expressive framework for modeling association rules. Unlike standard graphs, hypergraphs allow edges to connect multiple vertices simultaneously, making them well-suited for representing rules with multi-item antecedents. Jin et al. [5] model association rules as directed hypergraphs and propose a redundancy elimination procedure based on path-subsumption and structural analysis. Their approach transforms rule reduction into a graph problem, enabling the identification of subordinate and repetitive patterns. While theoretically elegant, this method has been empirically validated only on relatively small datasets, leaving questions regarding its scalability and applicability in real-world retail settings.

A parallel line of research shifts the focus from rule enumeration to the structural topology of product relationships. Instead of analyzing large sets of rules, network-based approaches model products as nodes and their associations as edges, enabling the study of global structure and interdependencies. Valle et al. [8] propose a framework in which product relationships are derived from correlations of binary purchase vectors and transformed into distance measures. By extracting a minimum spanning tree, the authors obtain a simplified representation of the product network that preserves the most important relationships while removing weaker connections. This approach facilitates the identification of product clusters, central items, and cross-category links that remain difficult to detect using traditional rule-based methods.

Despite their respective strengths, these two streams of research exhibit important limitations when considered independently. Hypergraph-based methods provide a principled way to represent and reduce association rules but have limited empirical validation in large-scale environments. Conversely, network-based approaches offer valuable structural insights but typically rely on correlation-based measures and do not explicitly address rule redundancy prior to network construction. As a result, the connection between association rule strength and network representation remains indirect.

This gap highlights the need for an integrated approach that combines efficient rule reduction with structurally meaningful network extraction. Specifically, there is a lack of scalable methodologies that (1) effectively eliminate redundancy in large rule sets, (2) construct product networks based directly on association rule metrics, and (3) produce simplified yet interpretable representations of product relationships that retain the most relevant structural information.

The present study addresses this gap by proposing a hybrid framework that integrates a confidence-improvement rule pruning procedure with a Lift–Confidence weighted network projection and MaxST extraction. By combining these elements, the proposed approach aims to provide both computational efficiency and structural interpretability, enabling a more comprehensive analysis of consumer purchasing behavior in large-scale retail datasets.

## 3. Proposed approach

### 3.1. Data preparation and transformation

The initial dataset consists of individual purchase records containing the consumer identifier, transaction date, sales channel, and product category. Since a unique receipt identifier was not available, it was necessary to define an approximation of the transaction unit, i.e., the consumer basket.

A consumer basket was defined as the set of all products purchased by the same consumer on the same day through the same sales channel. Formally, the basket identifier is defined as: BasketID = f(CustomerID, Date, Channel)

Each basket represents a subset of the universal set of products:


$$\begin{array}{c} t_{i}\subseteq I \end{array}$$

where:

$t_{i}$– the *i*-th transaction (basket),

I– the set of all products.

The complete set of transactions is defined as:


$$\begin{array}{c} {T=\{t}_{\text{1}},t_{\text{2}},\ldots ,t_{n}\} \end{array}$$

In accordance with the standard transactional model used in association rule analysis [18,19], a transaction represents a set of products purchased in a single shopping event.

### 3.2. Generating association rules using Apriori algorithm

To identify relationships between products, the Apriori algorithm was applied, which is one of the most commonly used algorithms for MBA [20].

The Apriori algorithm generates association rules of the form:


$$\begin{array}{c} X\to Y \end{array}$$

where:


$$\begin{array}{c} X\subseteq I,Y\subseteq I,X\cap Y=\emptyset \end{array}$$

The quality and strength of the rules were evaluated using three standard measures: support, confidence, and lift.

Support represents the relative frequency of products appearing together:


$$\begin{array}{c} support(X\to Y)=\frac{|\{t_{i}\in T:X\cup Y\subseteq t_{i}\}|}{|T|} \end{array}$$

Confidence represents the conditional probability of the consequent occurring given the occurrence of the antecedent:


$$\begin{array}{c} onfidence(X\to Y)=\frac{support(X\cup Y)}{support(X)} \end{array}$$

Lift represents a measure of statistical dependence between products:


$$\begin{array}{c} ift(X\to Y)=\frac{confidence(X\to Y)}{support(Y)} \end{array}$$

The values of the Lift metric are interpreted as follows [10]:

- lift > 1 – positive association
- lift = 1 – independence
- lift < 1 – negative association

### 3.3. Concepts of graph and hypergraph theory

A graph is defined as an ordered pair G=(V,E), where V is the set of vertices and E⊆V×V is the set of edges connecting pairs of vertices [21]. Edges may be undirected or directed, depending on whether the relationship between vertices has an orientation.In the context of MBA, vertices represent product categories, while edges represent the existence of an association between product categories.

Although graphs allow modeling relationships between pairs of vertices, in many real systems relations involve more than two elements simultaneously. In such cases, standard graphs may be insufficient for an adequate representation of the data structure.

A hypergraph is a generalization of a graph and is defined as an ordered pair $H=(V,E_{H})$, where V is the set of vertices and $E_{H}\subseteq {\text{2}}^{V}$ is the set of hyperedges [22].

Unlike a standard graph, where each edge is an ordered or unordered pair of vertices, a hyperedge represents an arbitrary subset of vertices, i.e., $e\in E_{H}\Rightarrow e\subseteq V$, which means that a single hyperedge can connect more than two vertices simultaneously. For example, a hyperedge of the form e={A,B,C}, represents a relation that simultaneously connects all three vertices, whereas in a standard graph the same relation would need to be represented using three separate edges: (A,B),(A,C)and (B,C).

In this study, the hypergraph perspective is used only as a conceptual foundation: rules sharing the same consequent are treated analogously to hyperedges incident to a common vertex, enabling the identification of redundant rules through subset comparisons of their antecedents. The implemented method does not construct or analyze an explicit hypergraph structure; instead, it applies a confidence-improvement pruning procedure based on consequent grouping and antecedent-set comparisons.

### 3.4. Set-based interpretation of association rules

The concepts introduced in the previous section provide a useful basis for interpreting association rules as set-based relationships among product categories.

Let V denote the set of product categories. Each association rule consists of an antecedent item set X ⊆ V and a consequent item set Y ⊆ V, with X ∩ Y = ∅. Therefore, an association rule does not necessarily describe only a pairwise relationship between two individual products, but may express a relationship between subsets of the product space, analogous to a hyperedge.

In this study, the set-based view of association rules is used to motivate the organization of rules prior to redundancy reduction. Rules with the same consequent are treated as structurally comparable because they refer to the same target product category and differ only in the composition of their antecedent sets. This provides the conceptual basis for the confidence-improvement pruning procedure described in the following section, where rules sharing the same consequent are compared through subset relations among their antecedent sets.

### 3.5. Reduction of redundant rules

The Apriori algorithm often generates a large number of redundant rules that do not significantly contribute to the interpretation of the data. To address this issue, the generated association rules were grouped according to their common consequent, where each group represents a set of rules sharing the same right-hand side (RHS). Formally, for a given product Y, the subset of all rules having Y as the consequent was defined. Reduction was performed by comparing antecedent sets among rules with the same consequent, where more complex rules were removed if they did not provide sufficient improvement relative to simpler ones.

Rule reduction was performed using a confidence-improvement criterion, following the general idea of improvement-based pruning introduced by Bayardo and Agrawal [23].

For two rules:


$$\begin{array}{c} X\to Y \end{array}$$

and


$$\begin{array}{c} X\cup Z\to Y \end{array}$$

The improvement is defined as:


$$\begin{array}{c} Improvement=confidence(X\cup Z\to Y)-confidence(X\to Y) \end{array}$$

If Improvement < δ, where δ  =  0.05, the rule containing the expanded antecedent (i.e., a larger number of elements in the conditional part) is treated as redundant and eliminated from further analysis.

This procedure removes rules that do not provide a significant improvement in the predictive power of the model.

The improvement threshold δ controls the aggressiveness of redundancy reduction. Larger values of δ lead to stronger pruning by requiring a greater increase in confidence for an expanded rule to be retained. In the experiments, δ = 0.05 was adopted as a conservative intermediate baseline value. This threshold requires an expanded antecedent to increase rule confidence by at least five percentage points in order to be retained, which provides a transparent compromise between removing weakly improving redundant rules and avoiding overly aggressive pruning. The choice was not treated as an optimal universal value; rather, it was used as a baseline setting and its robustness was evaluated through sensitivity analysis, as reported in Section 5.

The rule reduction procedure is formalized in the following pseudocode (see Project Code Repository [24] for the full Python implementation). The project repository includes the confidence-based implementation used in the main pipeline, a configurable multi-metric version used for robustness comparison, and a small sample dataset intended for testing the execution pipeline. The full anonymized dataset used to obtain the results reported in this study is available in a separate Zenodo repository [25].

**Pseudocode: Rule Reduction**

Input: Set of rules R, threshold τ

Output: Reduced set of rules R_reduced

1. Group rules by conclusion (right-hand side):

for each rule r ∈ R:

add r to group G[r.conclusion]

2. Initialize set rules_to_remove = ∅

3. For each group G:

sort rules in G by increasing premise size

initialize kept_rules_by_size = empty map

for each rule r in G:

for each subset s ⊂ premise(r):

if s exists in kept_rules_by_size:

parent_conf = confidence(s → RHS)

if confidence(r) – parent_conf < τ:

mark r for removal

break

if r not marked:

update kept_rules_by_size with r

4. R_reduced = R \ rules_to_remove

Return R_reduced

The proposed algorithm removes rules whose extended antecedent does not provide a significant increase in confidence compared to their corresponding subsets.

In the redundancy reduction process, the confidence metric was used, as it directly reflects the predictive power of a rule, i.e., the probability of the consequent given the antecedent. This choice enables the elimination of rules whose extension of the antecedent does not lead to a meaningful improvement in predictive performance.

To assess the robustness of the reduction procedure, an additional experiment was conducted in which redundant rules were removed using three alternative improvement criteria: based on the confidence metric, based on the lift metric, and based on a combined metric (lift × confidence).

The experiment was carried out for parameter values maxlen = 3, 4, 5, 6, comparing both the number of retained rules and the structure of the resulting MaxST. This approach enabled the evaluation of how the choice of reduction metric affects model complexity and the stability of the network representation.

The empirical results presented in Section 5 confirm that reduction based on the confidence metric leads to a significantly greater decrease in the number of rules compared to lift and the combined metric, particularly for higher values of the maxlen parameter. This experiment explicitly validates the choice of the confidence-based reduction criterion and evaluates its impact on the resulting network structure.

### 3.6. Projection of the reduced rule set into a product graph

Since network analysis algorithms, including MaxST, operate on standard graphs, it was necessary to project the reduced rule set into a graph.

Each rule of the form {A,B}→C was transformed into edges: (A,C)  and (B,C). The weight of each edge was defined as a combination of the lift and confidence metrics:


$$\begin{array}{c} weight(A,C)=lift(A\to C)\times confidence(A\to C) \end{array}$$

This score is used as a pragmatic edge-ranking heuristic rather than as a formally derived or standard association-rule interestingness measure. The rationale for combining the two measures is that they capture complementary aspects of rule quality: lift reflects the degree to which the observed co-occurrence exceeds what would be expected under statistical independence, while confidence reflects the conditional reliability of the implication.

Multiplication was selected because it is monotonic in both components: for positive values of lift and confidence, an increase in either measure increases the resulting edge score, provided that the other component remains unchanged. In addition, the product penalizes rules that are strong on only one dimension but weak on the other, thereby favouring product links that are both statistically dependent and conditionally reliable. This property is useful for MaxST extraction, where the objective is to retain a compact set of high-priority edges in the projected product network.

Compared with alternative combinations, multiplication was selected because it provides a simple ranking rule without introducing additional modelling choices. A weighted sum would require assigning relative importance to lift and confidence. Giving greater weight to confidence would favour more reliable rules, but could also privilege less distinctive associations involving frequent consequents. Conversely, giving greater weight to lift would favour more statistically distinctive associations, but could reduce the emphasis on conditional reliability. Since there was no theoretical or empirical basis for preferring one dimension over the other, no explicit weighting scheme was introduced. A harmonic mean would also require prior normalization, because lift and confidence are measured on different scales. Although normalization is possible, it would introduce further methodological choices, including the normalization method and whether it is applied globally or separately for each experimental configuration. The product was therefore retained as a transparent heuristic that gives high priority only to rules that are simultaneously strong in terms of statistical dependence and conditional reliability.

The resulting score should not be interpreted as a standardized probability-based measure, a dimensionally homogeneous index, or a test statistic. Its role is limited to ranking candidate product-pair links before extracting the MaxST. This use is consistent with weighted network projection approaches, where edge weights are defined according to the analytical objective and then used to obtain a simplified representation of the most relevant links in the network [21,26].

If multiple rules exist between two nodes, the maximum edge weight is retained, thereby preserving the highest-scoring relationship between products.

Formally, the weight of the edge between nodes i  and j  is defined as:


$$\begin{array}{c} w_{ij}=\left\{ \begin{array}{c} \underset{r\in R_{ij}}{\text{max}}\left(lift(r)\cdot confidence(r)\right),\;R_{ij}\neq \text{0}\\ \text{0},\;R_{ij}=\text{0} \end{array}\right. \end{array}$$

where $R_{ij}$ denotes the set of all rules in which products i and j  appear in an antecedent–consequent relationship.

This projection implies a loss of information about higher-order relationships (e.g., the joint effect of multiple products in the antecedent), but it significantly simplifies the structure and enables the application of standard graph-based methods.

The graph construction procedure can be formally represented by the following pseudocode:

**Algorithm: Projected Graph Construction from Rules**

Input: Reduced rules dataset R

Output: Weighted undirected graph G = (V, E)

1. Initialize empty graph G

2. For each rule r ∈ R:

premises = split(r.premises)   # extraction of antecedent and consequent elements

conclusions = split(r.conclusion)

weight = r.lift × r.confidence   # computation of edge weight

3. For each item p ∈ premises:

For each item c ∈ conclusions:

if p == c:   # avoidance of self-loops

continue

if edge (p, c) already exists in G:

if weight > existing_weight(p, c):   # retention of the stronger connection

update edge (p, c) with:

weight = weight

lift = r.lift

confidence = r.confidence

else:

add edge (p, c) to G with attributes:    # addition of a new edge

weight = weight

lift = r.lift

confidence = r.confidence

4. Return G

### 3.7. Concepts of trees and spanning trees

A tree is a connected graph without cycles. Formally, a graph T=(V,E) is a tree if it is connected and contains no cycles [21].

For a graph with n  vertices, a tree contains exactly n−1  edges.

A spanning tree T of a graph G  is a subgraph that [21]:

- includes all vertices of the graph,
- contains no cycles,
- has the minimum possible number of edges.

A minimum spanning tree is a spanning tree that minimizes the total weight of its edges, while a MaxST maximizes this value [27,28].

In this study, due to the definition of edge weights, a MaxST is used.

### 3.8. Construction of the Maximum Spanning Tree

From the resulting graph, a MaxST was constructed. Since the edges were weighted using the composite score Lift × Confidence, the resulting tree preserves the globally highest-scoring product associations while maintaining connectivity across the network.

Formally, a MaxST is a subgraph that connects all nodes, contains no cycles, and maximizes the total sum of edge weights, i.e.,:


$$\begin{array}{c} MaxST=\text{arg}\underset{T\subseteq G}{\text{max}}\sum_{e\in T}weight(e) \end{array}$$

where:


$$\begin{array}{c} |T|=|V|-\text{1} \end{array}$$

The MaxST represents the minimal set of the highest-scoring relationships that connect the entire product system [21,27,28].

Algorithms for constructing spanning trees, such as Kruskal’s and Prim’s algorithm, represent standard methods for solving optimization problems in graph theory.

### 3.9. Identification of central products

To identify structurally important product categories in the MaxST, two centrality measures were used: degree centrality and normalized betweenness centrality [[29]].

The degree of a node captures the number of direct connections of a product category:


$$\begin{array}{c} deg(v)=\left|\{u\epsilon V:(u,v)\epsilon E\}\right| \end{array}$$

where deg(v) denotes the number of edges incident to node v. The corresponding normalized degree centrality is defined as:


$$\begin{array}{c} C_{D}(v)=\frac{deg(v)}{n-\text{1}} \end{array}$$

where n denotes the total number of nodes in the network. In this study, products with high degree and degree centrality are interpreted as local hubs, i.e., categories directly connected to many other product categories.

Normalized betweenness centrality captures the extent to which a product category lies on shortest paths connecting other product categories. For an undirected graph, it is defined as:


$$\begin{array}{c} C_{B}(v)=\frac{\text{2}}{\left(n-\text{1})(n-\text{2}\right)}\sum_{\begin{array}{c} s,t\in V\backslash v\\ s<t \end{array}}\frac{\sigma_{st}(v)}{\sigma_{st}} \end{array}$$

where σ_st denotes the total number of shortest paths between nodes s and t, and σ_st (v) denotes the number of those shortest paths that pass through node v. This measure is particularly relevant in the MaxST because some categories may have a relatively small number of direct links but still occupy important intermediary positions between product chains.

Since edge weights in this study represent association strength rather than distance, normalized betweenness centrality was computed on the unweighted MaxST topology. Therefore, degree centrality was used to identify local hubs, while normalized betweenness centrality was used to identify bridge categories that connect different parts of the product network.

## 4. Experimental setup

### 4.1. Data description and preparation

The dataset used in this research consists of raw transactional records obtained from a major Montenegrin retailer, one of the three largest retail chains in the country. Due to the volume of data, the retailer provided monthly files covering an 11-month period from February 2024 to December 2024, with offline and online transactions delivered separately. After preprocessing, 14,910,624 product records were used to construct 2,636,756 basket transactions, consisting of 2,631,713 offline and 5,043 online basket transactions. The original raw dataset contained 14,913,174 product-level records, of which 2,550 records were excluded during preprocessing because they could not be reliably assigned to valid basket transactions. Each transaction includes customer-level and product-level attributes: customer ID, gender, age, product category, product price and transaction date. Before analysis and public release, the original raw dataset was anonymized and pre-processed to retain only the variables required for market basket construction and association rule mining.

For the purposes of association rule mining, all monthly datasets were pre-processed and transformed into a market-basket structure with product subcategories and transactions converted to binary vector representation. In this format, each column corresponds to a subcategory, with a value of 1 indicating that the subcategory was purchased in the transaction and 0 indicating its absence.

Following the data-aggregation rationale of Valle et al. [8], where individual SKUs were consolidated into broader analytical product groups to reduce noise and sparsity, this dataset contained 46 product groups. Many items differed only in packaging size, brand or format, and therefore represented the same underlying product from a purchasing-behaviour perspective. This ensured that the dataset retained behavioural meaning while enabling robust extraction of frequent itemsets and association rules.

Conducting the analysis at a highly disaggregated SKU level would substantially increase sparsity, inflate the number of rare item combinations, and reduce the interpretability of the resulting rules and networks. The selected category-level grouping reflects the retailer’s operational category-management structure and enables results to be interpreted in a form that is directly relevant for promotional planning, assortment decisions, and shelf-layout analysis.

### 4.2. Research pipeline and implementation

**Fig 1:**
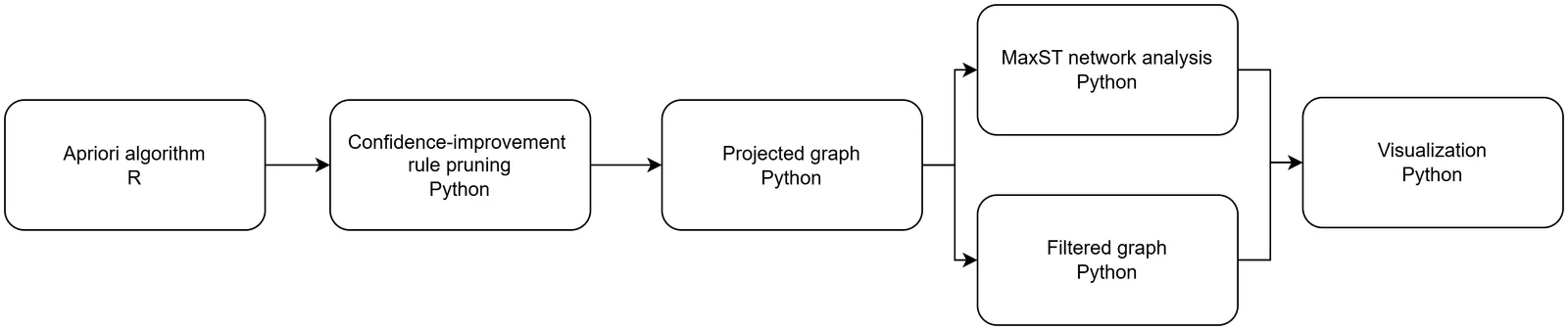
Rule reduction procedure.

The overall analysis follows a four-stage pipeline, as illustrated in Fig 1:

1. Generation of association rules using the Apriori algorithm.
2. Redundancy reduction of the generated rules using a confidence-improvement pruning procedure. This step groups rules sharing the same consequent and removes expanded antecedents that do not provide a sufficient improvement in confidence (δ = 0.05).
3. Projection of the reduced rules into a weighted undirected product graph, where the weight of each edge is defined as lift × confidence.
4. Extraction of the MaxST and construction of filtered graphs at different strength thresholds.

Association rule mining was performed in R using the arules package. Subsequent steps (rule reduction, graph construction, and network analysis) were implemented in Python using the Polars and NetworkX libraries.

The Apriori algorithm was executed with the following parameters:

- minimum support = 0.001
- minimum confidence = 0.3
- minimum lift = 1
- minimum/maximum rule length = 2/3 (additional experiments were conducted with maxlen = 4, 5, 6 and 7)

In the final reproducibility runs, the time-limit parameter in the arules implementation was disabled by setting it to 0, in order to avoid premature termination of rule generation for longer maxlen values.

The selected parameter values represent a compromise between rule quality, interpretability, and computational feasibility. The minimum support threshold of 0.001 corresponds to approximately 2,600 transactions in the analyzed dataset, ensuring that retained rules are supported by a substantial number of observations. The minimum confidence threshold of 0.3 was chosen to retain only rules with meaningful predictive strength, while the lift threshold of 1 eliminates associations that do not exceed random co-occurrence. Additional main-pipeline experiments with maxlen values of 4, 5, 6, and 7 were conducted to evaluate changes in the resulting product graphs and MaxST structures, while the extended sensitivity and Valle-type comparison analyses were reported for maxlen values up to 6.

As one of the main challenges was analysis of the large dataset in the initial steps of rule generation, R programming language was chosen due to its Apriori function and faster data manipulation and analysis. On the other hand, Python was used in subsequent steps due to its advantage in terms of graph libraries and visualisation possibilities. The experiments were executed on a laptop equipped with an Intel Core i5-1135G7 processor, 4 cores and 8 threads, 16 GB DDR4 RAM, and an NVMe SSD drive. GPU acceleration was not used in the analysis.

## 5. Results

### 5.1. Pipeline interpretation

The results of the analysis are based on a multi-stage approach that includes the generation of association rules, their reduction, and network representation using graph structures. The initial rule set obtained with the Apriori algorithm was further filtered and transformed into a product network, where edges were weighted using the combined measure lift × confidence. To facilitate interpretation, the analysis focused on the MaxST and on filtered graphs constructed using different thresholds of association strength.

The results show that some products are not included in the final rule set. These products either appear very rarely in transactions or do not form sufficiently strong associations with other products to satisfy the defined support and confidence thresholds. For example, certain categories occur only a few times within a dataset containing nearly 14.9 million product records and 2.64 million basket transactions, which prevents the formation of sufficiently supported association rules.

### 5.2. Rule reduction using confidence improvement

Applying the confidence-improvement pruning procedure defined earlier, a substantial reduction in the number of rules was achieved (from 4,677–3,127), while preserving informative relationships and removing a large number of redundant and overlapping rules.

An additional analysis was conducted to examine the impact of the reduction metric and maximum rule length on the number of retained rules. The results, presented in Table 1, reveal substantial differences across the applied criteria. The confidence-based criterion consistently produced the strongest reduction, particularly as the maximum rule length increased. The conducted experiment explicitly validates the choice of the confidence-based reduction criterion, demonstrating its effectiveness in balancing rule reduction and structural preservation.

**Table 1:** Number of retained association rules after reduction using different metrics and maximum rule lengths (maxlen).

Maxlen	Initial number of rules	Confidence-based	Lift-based	Lift × Confidence
3	4,677	3,127	4,435	4,403
4	32,173	16,619	29,002	29,434
5	142,170	52,965	116,421	124,025
6	403,817	137,186	294,558	333,245

To further assess the effect of the reduction metric on the downstream network structure, the resulting MaxSTs were compared across the three criteria. The choice of reduction metric affects the final network structure, although a substantial common core is preserved. For maxlen = 3, the confidence-based MaxST differed from the lift-based and Lift × Confidence-based MaxSTs by seven and eight edge substitutions, respectively. For maxlen = 4, 5, and 6, the lift-based and Lift × Confidence-based MaxSTs were identical, while the confidence-based MaxST differed from them by ten, nine, and nine edge substitutions, respectively (see supporting information S1 Table for details).

These findings indicate that the MaxST maintains a robust core of product relationships regardless of the reduction criterion, with differences primarily appearing in peripheral connections. This supports the stability of the proposed approach and justifies the use of the confidence-based criterion as the main reduction method.

To evaluate the sensitivity of the reduction procedure, the improvement threshold δ was varied from 0.01 to 0.10. As expected, the number of retained rules decreased monotonically from 4,583–1,349 as δ increased. The resulting trees retained 36 nodes and 35 edges throughout the examined range, but their edge composition was not invariant. Relative to the baseline tree at δ = 0.05, the number of shared edges ranged from 19 to 35. Delicatessen – Processed Meat Products remained the top hub for δ values from 0.01 to 0.08, whereas Sweets and Snacks became the top hub at δ = 0.09 and δ = 0.10. The analysis therefore indicates stability in tree size and moderate-threshold hub identity, but sensitivity in the detailed backbone topology. This supports the use of δ = 0.05 as a balanced baseline setting rather than as a universally optimal threshold (reported in S2 Table).

In addition to the sensitivity analysis for the confidence-improvement threshold δ, a separate sensitivity test was conducted for the minimum support and minimum confidence thresholds, while keeping maxlen = 3, lift = 1, δ = 0.05, confidence-based reduction, and Lift × Confidence MaxST weighting unchanged. The results are reported in S3 Table. Across the six additional scenarios, the number of initial rules ranged from 3,025–7,112, while the number of retained rules after confidence-based reduction ranged from 2,044–4,799. Despite these changes in rule volume, the key structural conclusions remained stable. The top hub was Delicatessen – Processed Meat Products in all scenarios, and a substantial part of the MaxST backbone was preserved. The MaxST shared between 23 and 34 edges with the baseline tree, depending on the threshold combination. The strongest structural stability was observed when only the confidence threshold was varied, with 34 shared edges at confidence = 0.20 and 32 shared edges at confidence = 0.40. Stricter support settings reduced the number of nodes from 36 to 34, indicating that some peripheral categories no longer satisfied the rule-generation thresholds, while the dominant hub and a substantial part of the backbone remained preserved.

As a supplementary statistical validation step, the retained association rules were evaluated using one-sided Fisher exact tests based on reconstructed basket-level 2 × 2 contingency tables. The tests were applied separately to each maxlen-specific retained rule set, and p-values were adjusted using the Benjamini–Hochberg false-discovery-rate procedure at the 5% level. The results, reported in S4 Table, show that almost all retained rules remained statistically significant after correction, indicating that the retained post-pruning rules are unlikely to be explained by random co-occurrence alone. This test directly evaluates the null hypothesis of independence between the antecedent and consequent of each retained rule, thereby providing a rule-level assessment of whether the observed associations could arise by chance. Although we did not estimate a full empirical null distribution of lift through permutation resampling, the corrected Fisher tests provide a formal significance assessment under the independence null model.

The execution-time results show that computational cost is dominated by data preparation rather than Apriori mining. For maxlen = 6, data preparation required 136.66 seconds, while Apriori mining required 12.75 seconds. Confidence-based rule reduction and graph processing remained comparatively fast, requiring 5.23 and 2.21 seconds, respectively, for the largest tested configuration. The total main-pipeline time increased from 127.13 seconds at maxlen = 3 to 161.86 seconds at maxlen = 6. These measurements refer to the rerun on the anonymized dataset and should be interpreted as hardware, and run-specific execution times, shown in Table 2.

**Table 2:** Execution time of the main analysis pipeline by maximum rule length (seconds).

maxlen	Data preparation	Apriori mining	Rule export	Apriori subtotal	Confidence-based reduction	Graph projection + MaxST	Total main pipeline
3	121.12	5.61	0.20	126.93	0.05	0.15	127.13
4	124.48	8.36	0.47	133.30	0.29	0.29	133.88
5	123.76	10.03	1.50	135.29	1.50	0.99	137.78
6	136.66	12.75	5.01	154.42	5.23	2.21	161.86

### 5.3. MaxST analysis

The MaxST represents a simplified network structure that preserves the highest-ranked relationships between products while using the minimum number of edges. The resulting tree, consisting of 36 nodes and 35 edges, enables the identification of key relationships within the system.

It can be observed that certain categories play a central role in the network, particularly products from the fresh meat and delicatessen group, which connect a large number of other categories. These products function as hubs, indicating their importance in the formation of consumer baskets.

The centrality results confirm that Delicatessen is the dominant hub in the baseline maxlen = 3 MaxST, with the highest degree and betweenness centrality. Fresh Meat also occupies an important structural position, combining a relatively high number of direct links with a strong bridging role. Beauty and Personal Care has fewer direct connections, but its high betweenness centrality indicates that it serves as an important bridge between product chains in the tree. Laundry and Cleaning Products also appears among the highest-betweenness categories, indicating a secondary bridge role in the baseline tree, as shown in Table 3.

**Table 3:** Product categories with the highest betweenness centrality in the baseline MaxST.

Rank	Product category	Degree	Degree centrality	Betweenness centrality
1	Delicatessen – Processed Meat Products	13	0.3714	0.8168
2	Fresh Meat (Counter / Unpackaged)	5	0.1429	0.5462
3	Beauty and Personal Care	2	0.0571	0.4807
4	Laundry and Cleaning Products	3	0.0857	0.2151
5	Party Supplies	2	0.0571	0.2084

**Fig 2:**
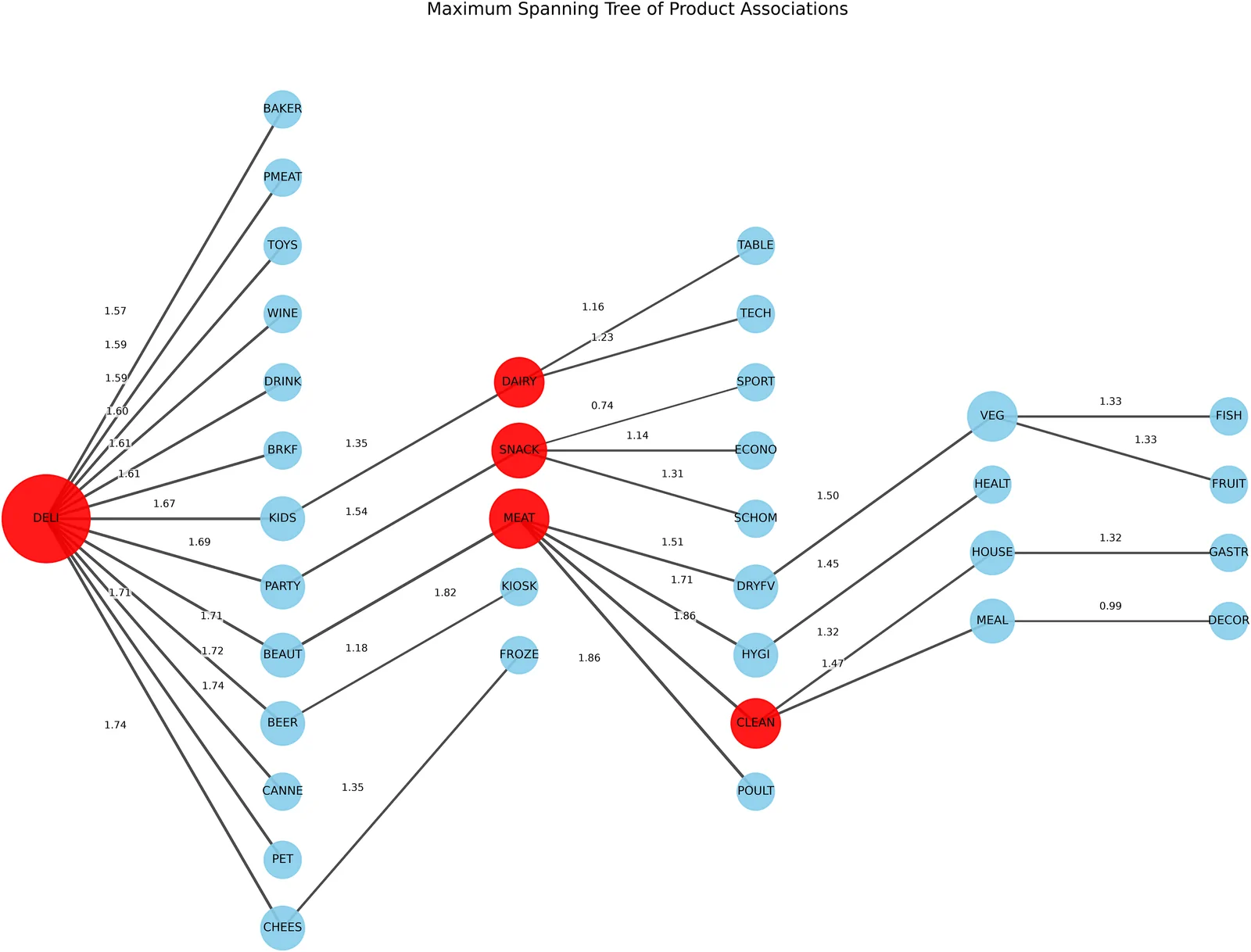
Maximum Spanning Tree of product associations. Node labels are abbreviated for readability. Full product category names corresponding to each abbreviation are provided in S5 Table. Red nodes represent the top five hubs.

The structure of the baseline tree (Fig 2) also reveals the presence of product chains, where some categories act as bridges between different product groups. The maximum distance from Delicatessen, used as the visualization root, is 5 edges, indicating moderate network depth and relatively short distances from the central hub to peripheral product categories.

The most prominent chains extracted from the MaxST are presented in Table 4.

**Table 4:** Selected product chains extracted from the MaxST (sorted by average edge weight – strongest first).

Rank	Chain Length	Product Chain	Avg. Weight per Edge	Business Insight
1	2	Delicatessen → Beauty and Personal Care → Fresh Meat	**1.76**	Cross-category association linking delicatessen, personal care, and fresh meat categories; suitable for testing bundle or placement strategies.
2	4	Delicatessen → Beauty and Personal Care → Fresh Meat → Dried Fruits and Vegetables → Vegetables	**1.63**	Meal-preparation oriented purchasing pathway connecting delicatessen, fresh meat, dried goods, and vegetables.
3	2	Delicatessen → Party Supplies → Sweets and Snacks	**1.62**	Celebration-related purchasing pattern linking delicatessen with party and sweets categories.
4	5	Delicatessen → Beauty and Personal Care → Fresh Meat → Dried Fruits and Vegetables → Vegetables → Fruits	**1.57**	Extended purchasing chain connecting delicatessen with personal care, fresh meat, dried goods, vegetables, and fruits.
5	3	Delicatessen → Children’s World → Dairy Products → Tableware / Kitchenware	**1.39**	Family-oriented purchasing pattern linking children’s products, dairy, and tableware categories.

Although the MaxST preserves the globally highest-scoring connections, its structure imposes certain limitations.

First, the MaxST maximizes the total weight of the tree under the constraint of acyclicity, which means that some strong connections may be excluded if an alternative path between the same nodes already exists. As a result, certain edges with high lift × confidence values are not included because their addition would create a cycle in the graph. In addition, the MaxST does not consist exclusively of the strongest edges. It also includes some weaker connections that are necessary to maintain the overall connectivity of the network. These edges typically connect peripheral products that have only a small number of associations with the rest of the system. Although some direct connections are not present in the MaxST, the relationship between those products is often preserved through indirect paths within the network, which partially retains the information about their association. As a consequence, the MaxST produces a simplified and hierarchical network structure, whereas the original graph clearly exhibits dense clusters of mutually connected products.

### 5.4. Filtered graphs analysis

In addition to the MaxST approach, filtered graphs were also analyzed. These graphs were constructed by retaining a selected percentage of the strongest edges in the network. The parameter FILTER_PERCENT determines the proportion of edges with the highest weights (lift × confidence) that are included in the graph. For example, a value of 20% means that only the top 20% strongest edges are retained, while increasing this parameter (e.g., to 40%) includes a larger number of edges, gradually adding relationships of decreasing strength.

**Fig 3:**
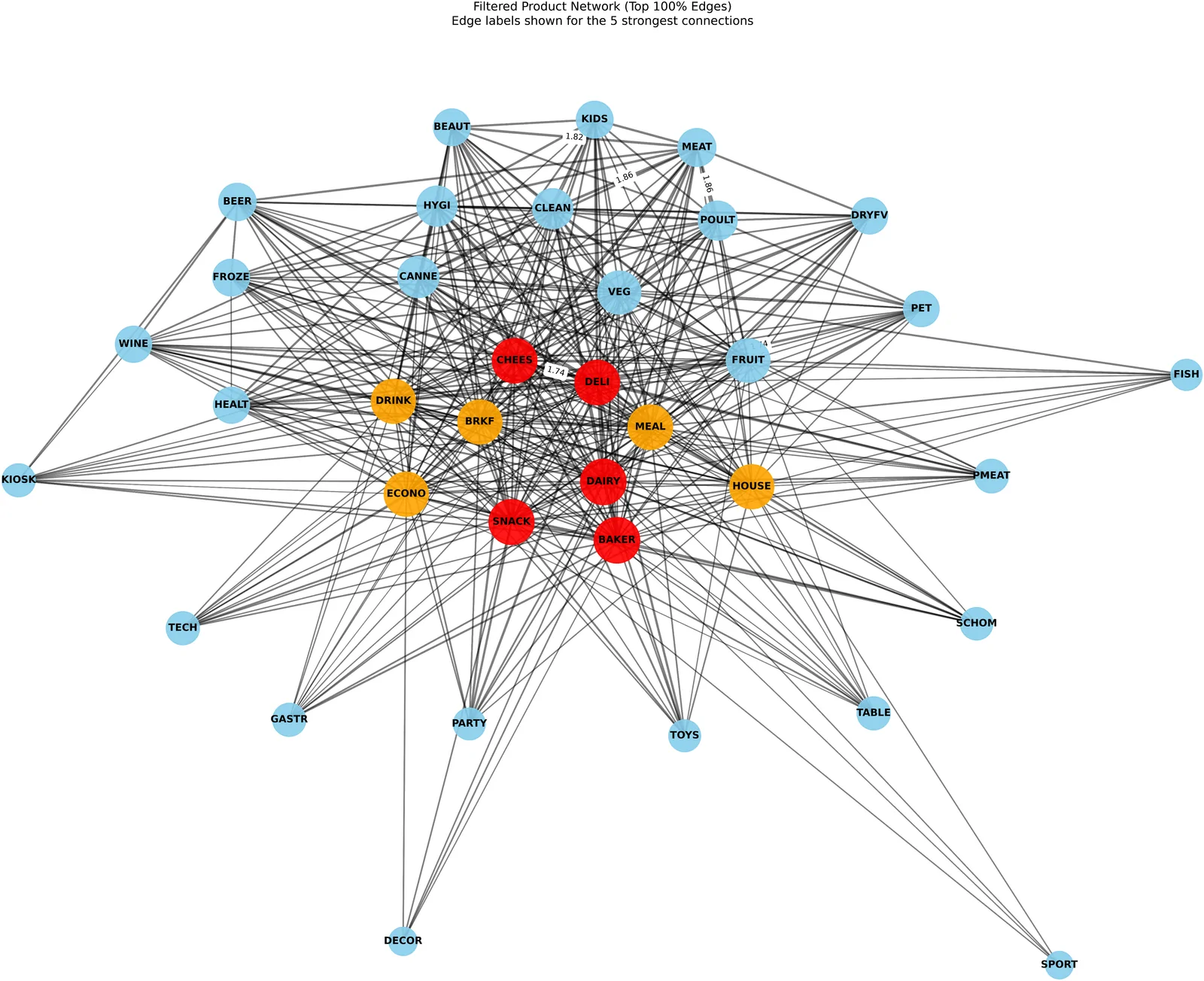
Filtered product network. Node labels are abbreviated for readability. Full product category names corresponding to each abbreviation are provided in S5 Table. Red nodes represent top 5 hubs, followed by orange as next 5. Edge labels identify the five projected product-pair edges with the highest Lift × Confidence weights.

When the parameter is set to 100%, the resulting graph contains all nodes and all edges generated during the association rule phase (Fig 3). It is important to note that this graph is not complete, but rather represents a reduced network obtained after applying the Apriori algorithm, where products and relationships that did not satisfy the minimum support and confidence thresholds were excluded from the analysis.

Unlike the MaxST, filtered graphs do not impose the acyclicity constraint, allowing cycles to exist and therefore preserving strong connections that may be omitted in the MaxST.

The five retained baseline rules with the highest lift are presented in Table 5. These rule-level results are distinct from the five projected product-pair edges labeled in Fig 3, which are ranked by Lift × Confidence.

**Table 5:** Five retained association rules with the highest lift in the baseline configuration.

Rank	Rule	Lift	Business Interpretation
1	Children’s World (Kids’ Products), Laundry and Cleaning Products → Beauty and Personal Care	4.47	Extremely strong association: customers purchasing children’s products and cleaning supplies also show a strong association with beauty/personal care purchases.
2	Hygiene and Paper Products, Laundry and Cleaning Products → Beauty and Personal Care	4.26	High-ranked co-purchase association between hygiene/paper, cleaning, and beauty categories.
3	Children’s World (Kids’ Products), Hygiene and Paper Products → Beauty and Personal Care	4.13	Family-oriented co-purchase pattern linking children’s products, hygiene, and beauty categories.
4	Non-Alcoholic Beverages, Wine and Spirits → Beer and Alcopops	4.09	High cross-category link between soft drinks, wine/spirits and beer/alcopops
5	Fresh Meat (Counter / Unpackaged), Pet Supplies → Poultry	4.04	Transactions containing fresh meat and pet supplies are strongly associated with poultry.

The five strongest interpreted rules reported in Table 5 were also subjected to a one-sided Fisher exact test based on basket-level 2 × 2 contingency tables. After Benjamini–Hochberg false-discovery-rate correction, all five rules remained statistically significant (adjusted p-values < 0.001), confirming that these high-ranked associations are unlikely to reflect random co-occurrence patterns. Thus, although a permutation-based expected lift distribution was not estimated, the strongest reported rules were formally tested against the independence null model and remained significant after correction for multiple testing.

### 5.5. Comparative and sensitivity analyses of network representations

A combined analysis of the MaxST and filtered graphs reveals the difference between the global and local importance of connections.

The MaxST identifies the minimal set of edges required to connect all products, providing a clear view of the global structure of the system. However, this comes at the cost of eliminating a large number of relationships, including some relatively strong connections. In contrast, filtered graphs provide insight into additional, locally significant relationships that are not included in the MaxST, including triangles and dense clusters of products. The presence of these structures indicates the existence of multiple and mutually interconnected purchasing patterns. Nodes that appear in the MaxST but are not connected in filtered graphs with lower percentage thresholds represent products whose associations are not among the top-ranked in the network. Their presence in the MaxST results from the need to maintain graph connectivity rather than from a dominant structural role. The difference between the MaxST and filtered graphs can therefore be interpreted as a trade-off between complexity reduction and information preservation, where the MaxST minimizes the number of edges, while filtered graphs allow a controlled increase in complexity.

The combined use of rule reduction based on confidence-improvement, MaxST extraction, and filtered graphs enables a multi-layered network analysis: from eliminating redundant rules, through identifying the global structure, to obtaining a detailed view of local connectivity patterns. This approach achieves a balance between reducing complexity and preserving key information, enabling reliable interpretation of consumer purchasing patterns and supporting informed business decision-making.

To further position the proposed approach relative to correlation-based product-network methods, an additional comparison was conducted using a Valle-type baseline [8]. In this baseline, product categories were represented as nodes, while edge weights were derived from φ/Pearson correlations computed from the basket-by-category binary matrix. A spanning-tree representation was then extracted to obtain a simplified correlation-based product network. The resulting correlation-based MST contained 46 nodes and 45 edges, with Laundry and Cleaning Products emerging as the top hub.

The comparison was performed on the 36 product categories shared with the proposed MaxST baseline (Table 6). The overlap between the two approaches was partial rather than complete. For maxlen = 3, the proposed MaxST shared 9 of 35 edges with the correlation-based MST (25.71%), while for maxlen = 4 the overlap increased slightly to 10 of 35 edges (28.57%). For maxlen = 5 and maxlen = 6, the overlap decreased to 4 of 35 edges (11.43%). Degree-rank correlations between the two network representations ranged from 0.4280 to 0.5164, indicating a moderate but not identical structural ordering of product categories.

**Table 6:** Comparison between the proposed Lift × Confidence MaxST and the Valle-type correlation-based MST.

maxlen	Shared edges	Edge overlap	Degree-rank correlation	Correlation-based MST hub	Proposed MaxST hub
3	9/35	25.71%	0.4880	Laundry and Cleaning Products	Delicatessen
4	10/35	28.57%	0.5164	Laundry and Cleaning Products	Delicatessen
5	4/35	11.43%	0.4280	Laundry and Cleaning Products	Beauty and Personal Care
6	4/35	11.43%	0.4353	Laundry and Cleaning Products	Beauty and Personal Care

These results show that the proposed Lift × Confidence MaxST and the correlation-based MST capture partially overlapping but distinct aspects of the product-association structure. The difference in central hubs is particularly informative: the correlation-based MST identifies Laundry and Cleaning Products as the dominant hub, whereas the proposed association-rule-based MaxST identifies Delicatessen as the dominant hub in the baseline maxlen = 3 configuration and Beauty and Personal Care in the longer-rule configurations. This difference is expected because the correlation-based MST is driven by marginal co-occurrence patterns across all categories, while the proposed approach first filters association rules through confidence-improvement pruning and then ranks projected product links using Lift × Confidence. The proposed method should therefore be interpreted as complementary to correlation-based spanning-tree approaches rather than as a direct replacement for them, with its practical value lying in the extraction of a compact rule-based backbone that prioritizes links supported simultaneously by statistical dependence and conditional reliability, without implying theoretical superiority or causal influence.

Hence, a combined analysis of the MaxST and filtered graphs reveals the difference between the global and local importance of connections, while the comparison with the Valle-type correlation-based MST further shows that the proposed approach captures distinct structural aspects relative to purely correlation-driven methods.

In addition to these comparisons, the sensitivity of the proposed MaxST to the choice of edge-weighting scheme was examined. To assess the effect of the edge-weighting scheme on the resulting backbone structure, a separate experiment was conducted using the same retained rule set (maxlen = 3, support = 0.001, confidence = 0.30, δ = 0.05), but with three alternative edge-weight definitions: Lift, Confidence, and Lift × Confidence. Three alternative edge-weight definitions were tested on this rule set: Lift alone, Confidence alone, and the proposed Lift × Confidence composite. All three projections were based on the same 3,127 retained rules and produced product graphs with 36 nodes and 379 edges. The resulting MaxSTs also had the same size, with 35 edges, but their edge composition differed substantially. The Lift-based MaxST shared only 6 of 35 edges with the proposed Lift × Confidence MaxST, while the Confidence-based MaxST shared 8 of 35 edges. This confirms that the MaxST topology is sensitive to the choice of edge-weighting scheme. The proposed Lift × Confidence weighting is therefore not interpreted as a universally optimal measure, but as a deliberate balanced ranking heuristic that prioritizes product links that are simultaneously statistically distinctive, as captured by Lift, and conditionally reliable, as captured by Confidence. The results are reported in S6 Table.

### 5.6. Result stability and practical implications

The main-pipeline analysis across different maximum rule-length settings shows that the resulting MaxST structure changes as maxlen increases. When maxlen was increased from 6 to 7, the number of initial rules rose from 403,817–769,580, while the number of retained rules increased from 137,186–209,176. The MaxST size remained unchanged, with 36 nodes and 35 edges, but only 24 of 35 edges were shared with the maxlen = 6 tree. This indicates stability in network size, but not full topological invariance, when longer rules are admitted.

The sensitivity analysis of minimum support and minimum confidence thresholds reported in Section 5.2 further confirms that the main structural conclusions are robust across alternative parameter settings: although the number of generated and retained rules changes, Delicatessen – Processed Meat Products remains the dominant hub in all tested scenarios, while a substantial part of the MaxST backbone is preserved. However, further lowering the support threshold would allow the generation of more complex rules, but this would simultaneously lead to a substantial increase in the number of rules, reduced interpretability, and increased computational cost.

The MaxST structure also demonstrates considerable stability with respect to the choice of rule reduction criterion. As shown in Section 5.2, although different improvement metrics lead to moderate differences in peripheral edges, the core topology and dominant hubs remain largely consistent. Also, moderate stability was observed regarding the improvement threshold δ (up to 0.08).

Additional segment-level results by gender, age group, and combined gender–age groups are reported in S7 Table. These results show that the dominant hub remains stable in the gender-only analysis and in most age groups, while combined gender–age segments reveal greater heterogeneity in the resulting MaxST structures.

Although the full dataset comprises 2,636,756 operationally defined baskets, the combined gender–age segmentation necessarily produces smaller and potentially unequal subsets. The greater heterogeneity observed in these groups may therefore reflect genuine demographic differences in purchasing patterns, but it may also partly reflect reduced support for less frequent associations and greater instability of peripheral MaxST edges. Accordingly, the segment-level results should be interpreted as exploratory indications rather than as conclusive evidence of demographic purchasing effects. Future research could assess their stability through segment-size reporting, resampling, customer-level holdout validation, or replication across independent time periods.

The results suggest that certain product categories play a central role in basket formation and can serve as anchors for promotions or cross-selling strategies. At the same time, products that remain isolated or weakly connected may require a different approach, such as targeted promotion or assortment reorganization.

Products with the highest purchase frequency (e.g., dairy, bakery, snacks) are not necessarily central in the network, whereas categories such as fresh meat and delicatessen occupy structurally important positions. This highlights the distinction between purchase frequency and structural importance within the network.

## 6. Discussion of results

### 6.1. Structural insights and key findings

The obtained results indicate a complex but stable structure of relationships between product categories within the analyzed system. By combining association rules with network analysis, key purchasing patterns were identified, with certain categories clearly emerging as central nodes connecting a large number of other products.

One of the most important findings is that the most frequently purchased products are not necessarily the most influential in the network structure. For example, although categories such as dairy products, bakery products, and snacks dominate in terms of transaction frequency, their role in the network is not as central as that of categories such as fresh meat or delicatessen. These categories act as key connectors between different product groups, indicating their strategic importance in the formation of consumer baskets.

Furthermore, the analysis of the MaxST shows that the network structure can be significantly simplified without losing the basic connectivity of the system. This interpretation is consistent with the constraint discussed in Section 5.3, where global connectivity is prioritized over retaining every strong local edge. This confirms that the MaxST provides a globally optimal but locally reduced representation of product relationships.

In contrast, filtered graphs preserve local patterns, including clusters and triangles of products that indicate alternative paths of association. The presence of these structures suggests that certain products may be linked through multiple purchasing patterns, which cannot be fully captured by the MaxST due to its acyclic constraint.

An important aspect of the analysis concerns products that are not included in the final network. Their absence does not necessarily imply irrelevance, but rather that they do not form sufficiently strong or frequent associations with other products within the observed transactions. Such products may represent specific or niche categories that require a different analytical approach and different business strategies.

The results also show that the MaxST structure continues to change when longer rules are admitted. Although increasing maxlen from 6 to 7 did not change the size of the MaxST, which remained at 36 nodes and 35 edges, the two trees shared only 24 of 35 edges. This suggests that longer rules can still affect the detailed backbone topology, even when the overall network size remains stable.

The sensitivity analysis of the rule reduction criteria further confirms the robustness of the proposed framework. Although the choice of improvement metric (confidence, lift, or Lift × Confidence) influences the exact composition of the MaxST, particularly at higher maximum rule lengths, a substantial common core of relationships is consistently preserved across all variants. Differences are mainly confined to peripheral edges, while the dominant hub (Delicatessen in the baseline configuration) and the overall network topology remain stable.

This moderate sensitivity is expected and even desirable: it demonstrates that the method does not depend critically on a single arbitrary choice, yet still allows the researcher to select the most appropriate reduction criterion depending on the desired balance between aggressiveness of pruning and retention of potentially valuable associations. The superior performance of the confidence-based criterion in reducing rule volume, combined with its limited impact on the core network structure, validates its selection as the primary reduction approach in this study.

The edge-weighting sensitivity analysis further shows that the selected weighting scheme is an important analytical choice for the detailed MaxST topology and its substantive interpretation. Although the Lift-, Confidence-, and Lift × Confidence-based MaxSTs had the same number of nodes and edges, their edge composition and dominant hubs differed substantially. Lift-based weighting gives priority to statistically distinctive associations, whereas Confidence-based weighting emphasizes conditional reliability. The proposed Lift × Confidence score represents a balanced heuristic that requires strength on both dimensions, but it should not be interpreted as a universally optimal or weight-invariant criterion. Practitioners should therefore select the weighting scheme according to the analytical objective and compare alternative MaxSTs when managerial conclusions depend on particular edges, chains, or hubs.

Similarly, the confidence-improvement threshold δ should be calibrated for the specific dataset and retail context rather than transferred mechanically across applications. In the present dataset, δ = 0.05 provided a balanced reduction baseline, and the dominant hub remained stable up to δ = 0.08; however, the changes observed at δ = 0.09 and δ = 0.10 indicate that stronger pruning can alter both the detailed backbone topology and its interpretation. Future applications should therefore examine a range of δ values and select a setting that provides an appropriate trade-off between rule reduction, structural stability, and the intended analytical purpose.

From a methodological perspective, the combination of rule reduction based on confidence-improvement, network representation, and graph algorithms proved to be an efficient approach for processing large transactional datasets. Importantly, the initially complex rule set was reduced to an interpretable structure without losing essential information, enabling clearer analysis and supporting decision-making.

### 6.2. Business implications

The network structure revealed in this study offers several empirically grounded indications that may inform retail strategy and operations and can be further tested in applied retail settings. The product chains identified in the MaxST (Table 4) and the top 5 ranked associations in the filtered product network (Table 5) highlight recurring purchasing patterns and potentially relevant cross-category relationships that may inform hypotheses for cross-category promotions, bundling, and assortment decisions.

The highest-scoring associations in the filtered product network (Table 5) consistently link family-oriented and household-maintenance categories with Beauty and Personal Care. These high-lift rules suggest a possible “family self-care” purchasing pattern, in which customers who buy children’s products, laundry and cleaning supplies, and hygiene items are also more likely to purchase beauty and personal care products. Such patterns may indicate opportunities for higher basket-value combinations, although their commercial impact should be empirically tested in a store or campaign setting. Additional high-ranked relationships suggest possible co-purchasing patterns in the beverage category and between fresh meat/poultry and pet supplies.

Complementing these findings, the baseline maxlen = 3 MaxST (Table 4) identifies Delicatessen as the central structural hub in the product association network. It appears in all five selected chains, most notably in the highest-ranked chain Delicatessen → Beauty and Personal Care → Fresh Meat, with an average edge weight of 1.76. This association may indicate a cross-category purchasing pathway linking delicatessen, personal care, and fresh meat categories. The longer chains further extend this pathway toward dried fruits and vegetables, vegetables, and fruits, suggesting a broader meal-preparation and healthy-produce pattern that could be further tested through bundle or shelf-layout experiments. Other chains link Delicatessen with Party Supplies and Sweets and Snacks, suggesting a celebration-related association, and with Children’s World, Dairy Products, and Tableware / Kitchenware, indicating a family-oriented purchasing pattern.

These insights suggest several practical applications that retailers could test:

- Developing targeted family bundles and “family shopping” promotions that combine children’s products, laundry/hygiene items and beauty & personal care products.
- Testing whether beverage bundles linking beer/alcopops with wine and soft drinks generate stronger cross-category purchasing than standard category-level promotions.
- Designing testable bundles centered on delicatessen that follow the selected purchasing chains, particularly those combining delicatessen with beauty and personal care, fresh meat, vegetables, fruits, and dried goods.
- Experimenting with shelf-layout alternatives that position strongly associated categories in closer proximity, such as beauty near children’s and cleaning aisles, beer near wine and non-alcoholic beverages, or delicatessen near fresh meat.

Overall, these results suggest the value of exploring a more relationship-based view of category management. However, the identified associations should be interpreted as empirically grounded hypotheses for managerial testing rather than as evidence of realized commercial effects. By using these associations to design pilot cross-category promotions, bundle offers, and shelf-layout experiments, retailers can test whether the observed patterns are associated with improved cross-selling performance, promotional effectiveness, or customer experience.

## 7. Limitations and future research

Although the results reveal stable and interpretable patterns, several limitations should be considered.

First, the analysis was performed at the level of retailer-defined product categories rather than individual SKUs or finer subcategories. This reflects the structure of the data provided by the retailer, which were extracted from its internal databases according to its operational category-management system. While this level of aggregation reduces sparsity, limits the number of rare item combinations, improves interpretability and managerial relevance of the resulting rules and networks, it may also introduce aggregation bias. Specifically, strong associations that exist only between particular products or subcategories may be weakened when grouped into broader categories, while some category-level associations may appear stronger because heterogeneous products are grouped together. Hence, the identified relationships should be interpreted as category-level purchasing patterns rather than product-level associations. Even though the available data did not support a systematic sensitivity analysis at a finer level of granularity, future research could examine whether the identified patterns remain stable when subcategory- or SKU-level data are available.

Although supplementary statistical validation was conducted using one-sided Fisher exact tests with Benjamini–Hochberg false-discovery-rate correction, the validation was focused on retained association rules and the strongest interpreted rules rather than on every possible rule generated during the exploratory mining process. In addition, no permutation- or bootstrap-based validation of the MaxST topology was conducted in the present study. Such validation would require repeated execution of the full analytical pipeline, including transaction-level resampling, Apriori rule generation, confidence-improvement pruning, graph projection, and MaxST extraction, which would be computationally demanding given the scale of the dataset and the large number of generated rules. Moreover, the MaxST is used in this study as a descriptive backbone-extraction tool rather than as a standalone inferential model; therefore, formal statistical validation was applied at the level of retained association rules, while network robustness was assessed through sensitivity analyses. Confidence intervals were not estimated for the reported rule and network metrics because they would quantify uncertainty around individual estimates but would not directly address the multiple-testing problem arising from the large number of generated candidate rules. For this reason, corrected significance testing using Benjamini–Hochberg false-discovery-rate adjustment was considered a more direct response to the statistical-validation concern. A separate holdout validation was also not conducted, because the objective of the study was exploratory structural discovery rather than predictive model evaluation; nevertheless, future work could examine the stability of retained rules and MaxST edges across independent temporal or customer-level holdout samples. Because the analysis relies on observational transaction data, the identified product relationships should still be interpreted as associative rather than causal.

Another limitation arises from the use of the MaxST, which removes cycles and therefore eliminates some information about multiple relationships between products. Although filtered graphs partially compensate for this limitation, a trade-off between complexity and completeness remains.

In addition, channel-based and seasonal analyses were not included in the present study due to data-balance and time-coverage limitations. Although the dataset contains channel information, the online channel accounts for only a very small fraction of the available records compared with offline purchases, which would make a direct comparison of online and offline product networks unstable and potentially misleading. Similarly, the available observation period covers February to December 2024, meaning that a complete winter season is not observed. For this reason, seasonal conclusions were not drawn. From an implementation perspective, however, the proposed pipeline can be readily adapted to channel-specific or season-specific subsets when more balanced channel data or complete multi-year seasonal data are available.

Possible directions for future research include:

- applying additional rule interestingness measures (e.g., conviction, leverage),
- using community detection algorithms in product networks,
- analyzing the temporal evolution of the network,
- developing interactive visualizations for practical use in business decision-support systems.

## 8. Conclusion

This study shows that large-scale market basket analysis can be made more interpretable when rule redundancy reduction and product-network extraction are treated as complementary stages of analysis. By integrating confidence-improvement rule pruning with a Lift × Confidence-weighted maximum spanning tree, the proposed approach significantly reduces the number of rules while preserving the core structure of product interdependencies.

Applied to a real-world dataset of approximately 2.64 million basket transactions from a major Montenegrin retailer, the methodology demonstrated substantial rule reduction under confidence-based pruning without compromising the interpretability of the resulting product network. The extracted MaxST revealed a robust core of relationships, with categories such as delicatessen, fresh meat, and beauty occupying structurally important positions rather than simply reflecting the most frequently purchased items (e.g., dairy or bakery). These findings highlight the importance of moving beyond simple frequency toward network centrality when designing cross-selling and assortment strategies.

The framework offers three main contributions: (1) a practical and computationally efficient procedure for redundancy reduction applied to large-scale retail transaction data, (2) a novel rule-strength-based MaxST that directly leverages association metrics instead of pairwise correlations, and (3) empirical validation on a large-scale, real-world setting rarely seen in the literature.

From a managerial perspective, the identified product chains and strong cross-category links provide empirically grounded hypotheses for bundle promotions, store layout optimization, and targeted marketing, particularly around meal-occasion assortments built around the Delicatessen hub identified in the baseline MaxST.

Future research could extend this work by incorporating temporal dynamics, customer-segment analysis, individual SKU-level analysis, community detection, or more advanced interestingness measures. To conclude, the approach offers a practical route from large, difficult-to-interpret rule sets to compact product networks that support retail decision-making.

## Supporting information

S1 Table Differing MaxST edges across reduction criteria and maximum rule-length settings. The table reports product-pair edges that appear only in one MaxST when comparing confidence-based, lift-based, and Lift × Confidence-based rule reduction criteria for maxlen values from 3 to 6.
(XLSX)

S2 Table Sensitivity of the baseline MaxST to the confidence-improvement threshold δ. This table reports the effect of varying δ from 0.01 to 0.10 (baseline: maxlen = 3, support = 0.001, confidence = 0.30, lift = 1, confidence-based reduction, Lift × Confidence weighting). For each δ value, the table shows the number of retained rules, MaxST size, shared edges relative to the δ = 0.05 baseline, number of different edges, and the top hub. The results indicate stable tree size (36 nodes, 35 edges) but moderate changes in edge composition and hub identity at higher δ values.
(XLSX)

S3 Table Sensitivity analysis of support and confidence thresholds. The table reports the number of initial and retained rules, MaxST size, overlap with the baseline MaxST, and the top hub for alternative support and confidence settings (baseline: support = 0.001, confidence = 0.30, maxlen = 3, lift = 1, δ = 0.05, confidence-based reduction, Lift × Confidence weighting).
(XLSX)

S4 Table Statistical validation of retained association rules. The table reports one-sided Fisher exact tests based on reconstructed 2 × 2 contingency tables for retained rules across maxlen values 3–6, including the five top-ranked rules from Table 5. P-values were adjusted using the Benjamini–Hochberg procedure at the 5% level.
(XLSX)

S5 Table Complete list of product categories and abbreviations used in the network figures. (XLSX)

S6 Table Sensitivity of the baseline MaxST to alternative edge-weighting schemes. The table compares Lift, Confidence, and Lift × Confidence weighting using the same retained rule set (maxlen = 3, support = 0.001, confidence = 0.30, δ = 0.05). It reports graph and MaxST sizes, shared edges relative to the proposed weighting, and the top hub.
(XLSX)

S7 Table Segment-level analysis by gender, age group, and combined gender–age groups. The table reports the number of initial and retained rules, MaxST size, overlap with the baseline, and the top hub for each segment (baseline configuration).
(XLSX)
